# Expression profiling and functional analysis reveals that TOR is a key player in regulating photosynthesis and phytohormone signaling pathways in *Arabidopsis*

**DOI:** 10.3389/fpls.2015.00677

**Published:** 2015-09-07

**Authors:** Pan Dong, Fangjie Xiong, Yumei Que, Kai Wang, Lihua Yu, Zhengguo Li, Maozhi Ren

**Affiliations:** School of Life Sciences, Chongqing UniversityChongqing, China

**Keywords:** TOR signaling, asTORis, AZD8055, gene expression profile, photosynthesis, phytohormone

## Abstract

Target of rapamycin (TOR) acts as a master regulator to control cell growth by integrating nutrient, energy, and growth factors in all eukaryotic species. TOR plays an evolutionarily conserved role in regulating the transcription of genes associated with anabolic and catabolic processes in *Arabidopsis*, but little is known about the functions of TOR in photosynthesis and phytohormone signaling, which are unique features of plants. In this study, AZD8055 (AZD) was screened as the strongest active-site TOR inhibitor (asTORi) in *Arabidopsis* compared with TORIN1 and KU63794 (KU). Gene expression profiles were evaluated using RNA-seq after treating *Arabidopsis* seedlings with AZD. More than three-fold differentially expressed genes (DEGs) were identified in AZD-treated plants relative to rapamycin-treated plants in previous studies. Most of the DEGs and Kyoto Encyclopedia of Genes and Genomes (KEGG) pathways involved in cell wall elongation, ribosome biogenesis, and cell autophagy were common to both AZD- and rapamycin-treated samples, but AZD displayed much broader and more efficient inhibition of TOR compared with rapamycin. Importantly, the suppression of TOR by AZD resulted in remodeling of the expression profile of the genes associated with photosynthesis and various phytohormones, indicating that TOR plays a crucial role in modulating photosynthesis and phytohormone signaling in *Arabidopsis*. These newly identified DEGs expand the understanding of TOR signaling in plants. This study elucidates the novel functions of TOR in photosynthesis and phytohormone signaling and provides a platform to study the downstream targets of TOR in *Arabidopsis*.

## Introduction

Target of rapamycin (TOR) is a Ser/Thr protein kinase that was first isolated in budding yeast (*Saccharomyces cerevisiae*) (Heitman et al., 1991), and then identified in animals and plants (Chiu et al., 1994; Sabatini et al., 1994; Menand et al., 2002). TOR is functionally and structurally conserved from yeast to plants and animals (Wullschleger et al., 2006). The TOR protein is comprised of the following five domains, in order from the N terminus to the C terminus: HEAT repeats, FAT, FRB, kinase, and FATC domains. In yeast and animals, the TOR protein resides in two complexes: rapamycin-sensitive TOR complex 1 (TORC1) and rapamycin-insensitive TOR complex 2 (TORC2) (Loewith et al., 2002; Wullschleger et al., 2006). The core members of TORC1 include TOR, lethal with SEC13 protein 8 (LST8) and regulatory-associated protein of TOR (RAPTOR). TORC1 controls cell proliferation and temporal growth by dynamically maintaining the homeostatic balance between anabolic and catabolic processes (Wang and Proud, 2009; Xiong and Sheen, 2014). TORC2, which mainly contains TOR, LST8, stress-activated map kinase-interacting protein 1 (SIN1) and rapamycin insensitive companion of TOR (RICTOR), regulates spatial cell growth by modulating the cytoskeleton structure, cell polarity, glycolysis, glycogenesis, lipogenesis, and gluconeogenesis (Loewith et al., 2002; Takahara and Maeda, 2013; Xiong and Sheen, 2014). The core members of TORC1 are highly conserved from the last eukaryotic common ancestor to humans, but those of TORC2 are more variable. For example, the homologs of the core components of TORC2 in animals, such as SIN1 and RICTOR, are missing in plants (Xiong and Sheen, 2014).

The disruption of TOR function has been lethal in all examined eukaryotic organisms, which has prevented definition of the TOR functions (Barbet et al., 1996; Zhang et al., 2000; Weisman and Choder, 2001; Menand et al., 2002; Murakami et al., 2004; Ren et al., 2011). Progress in this respect was not made until the discovery of rapamycin, which can repress TORC1 activity in yeast and animals very efficiently (Heitman et al., 1991; Chiu et al., 1994; Sabatini et al., 1994). However, rapamycin inhibits the activity of TORC1 only in the presence of 12-kDa FK506 binding protein (FKBP12) through forming a ternary complex rapamycin-FKBP12-TOR in yeast and animals (Benjamin et al., 2011). Many downstream effectors in the TOR pathway have been identified in yeast and animals, but little is known about them in plants because of general planta-wide insensitivity to rapamycin. Although plants do have the homologs of yeast or mammal FKBP12, they have evolved to be incompatible with rapamycin and TOR, and thus the rapamycin/FKBP12/TOR ternary complex cannot form properly in plants (Xu et al., 1998; Menand et al., 2002; Sormani et al., 2007). Interestingly, yeast and human FKBP12s could rescue rapamycin sensitivity in *Arabidopsis*, indicating that TORC1 is conserved sufficiently across eukaryotic organisms (Mahfouz et al., 2006; Sormani et al., 2007; Leiber et al., 2010; Ren et al., 2012; Xiong and Sheen, 2012; Zhang et al., 2013). The overexpression and RNAi of TOR in *Arabidopsis* were created to further decipher the TOR signaling pathway in plants (Deprost et al., 2007; Caldana et al., 2013). However, all these studies were dependent on transgenics, which severely limit the investigations of TOR in less developed plant models.

Ren et al. (2012) performed RNA-seq to examine the transcriptional changes after TOR repression by rapamycin in the transgenic *Arabidopsis* lines overexpressing yeast *FKBP12* for 3 days (Ren et al., 2012). Caldana et al. (2013) found DEGs by silencing TOR expressing in *amiR-tor* mutants for 3 or 6 days with the method of Microarrays (Caldana et al., 2013). The huge overlapping DEGs found in the above two studies drew similar conclusions, such as regulating the cell wall restruction, while, unexpectedly, their transcription profiles did not change significantly when TOR expression was suppressed (Ren et al., 2012; Caldana et al., 2013). In fact, only 271 DEGs were displayed between RNAi plants and their controls within 3 days of TOR suppression (Caldana et al., 2013). A possible reason for this was that Ren et al. (2012) and Caldana et al. (2013) harvested seedlings for transcriptional profiling after the repression of TOR at 3 or 6 days, and these time points could be too late to detect the early molecular events of TOR suppression (Ren et al., 2012; Caldana et al., 2013). Another possible reason is that the *in vivo* inhibition spectrum of rapamycin is narrow and mainly targets the TORC1-S6K signaling branch (Ren et al., 2012). Xiong et al. (2013) found more than 2000 DEGs at a photoautotrophic transition checkpoint in 3 days after germination (DAG) WT and RNAi seedlings with or without 2 h glucose induction (Xiong et al., 2013). However, in this study, the *Arabidopsis* seeds were germinated in liquid medium, which might have caused oxygen stress, and thus a knockdown of TOR kinase activity. Importantly, the accumulated evidence showed that auxin and hormone signaling were closely interconnected with TOR signaling, and the repressing or silencing of the TOR gene expression resulted in severe defects in chloroplasts and photosynthesis (Ren et al., 2011; Caldana et al., 2013; Schepetilnikov et al., 2013). However, the transcriptional profiling of the phytohormone signaling pathways and photosynthesis-associated genes did not show significant changes in these studies (Ren et al., 2012; Caldana et al., 2013; Xiong et al., 2013).

In order to overcome the limitations of previous TOR studies, the second generation of TOR inhibitors asTORis have been well-developed in mammalian systems (Apsel et al., 2008; Janes et al., 2010; Zhang et al., 2011) and were employed in our study. asTORis can selectively and efficiently suppress both TORC1 and TORC2 by specifically targeting the ATP-binding pocket of the TOR kinase domain (Feldman et al., 2009; Dowling et al., 2010). Recently, asTORis were successfully applied to inhibit TOR activity in flowering plants, including *Arabidopsis, Oryza sativa* (rice), *Panicum miliaceum* (millet), and *Lotus japonicus* etc., and micromolar concentrations were sufficient to suppress TOR activity and obtain a physiological response (Montané and Menand, 2013; Schepetilnikov et al., 2013; Xiong et al., 2013). In *Arabidopsis*, asTORis inhibited the level of root growth dependent on the number of copies of the AtTOR gene through a genetic method, and the specificity of TORIN1 for TOR kinase activity was confirmed by Western blot (Montané and Menand, 2013; Schepetilnikov et al., 2013; Xiong et al., 2013). Although the previous studies showed that asTORis provide a highly inducible, selective, and reversible system to characterize TOR signaling in plants (Montané and Menand, 2013; Xiong et al., 2013), little information is known about the transcription profile of plants treated with asTORis. In this study, we performed expression profiling and used a functional analysis to reveal the functions of TOR in the post-seedling stage in *Arabidopsis*. The detected DEGs support the evolutionarily conserved TOR function of ribosome biogenesis, autophagy, and cell growth from yeast to animals and plants (Wullschleger et al., 2006; Xiong and Sheen, 2014), and reveal some novel and unique functions of TOR in photosynthesis and phytohormone signaling in plants.

## Materials and methods

### Plant materials and growth conditions

*Arabidopsis thaliana* L. (Columbia ecotype) seeds were sterilized using a freshly prepared solution containing 10% sodium hypochlorite and 0.01% Triton X-100 for 5 min, and then washed five or six times with distilled water. The seeds were placed in a beaker with distilled water and vernalized at 4°C for 2 days, and then maintained at 22°C under white light for 8 h. All the growth experiments were conducted under the conditions of 22°C, 16 h light/day, and 100 mmol/m^2^/s fluorescence bulbs light.

### Measurement of the inhibitory effects of asTORis on *Arabidopsis*

AZD, TORIN1, and KU were selected for the inhibitor screening experiment, because they were the most selective inhibitors of TOR and they could represent the strong, moderate, and mild asTORis according to their 50% growth inhibition doses (GI50). The GI50 of AZD, TORIN1 and KU is 0.03–0.1 nM, 0.25 nM, and 2.5–10 nM, respectively. The prepared seeds were incubated on 0.76% agar plates containing half-strength Murashige-Skoog (0.5 × MS) nutrient medium, 1% sucrose and different types of asTORis (AZD, TORIN1, and KU, Bioshop Canada) (all the asTORis were dissolved in DMSO) at varying concentrations (0.5, 1, 2, 5, and 10 μM), with the same amount of DMSO in the controls (Fisher ChemAlert). On the 10th day after germination, the seedlings were photographed next to a ruler. The number of plants with green cotyledons was counted and the cotyledon greening rates in each plate were calculated (number of plants with green cotyledons/total number of plants). Their primary root lengths and shoot fresh weights were measured using ImageJ software and an electronic balance, respectively. In addition, plants grown for 10 days without drugs or DMSO in the plates containing 0.5 × MS and 1% sucrose were transferred to the same plates with inhibitors (0.5, 1, 2, 5, and 10 μM) or DMSO as controls, for 5 days. Then, the same measurements were collected as described earlier. For the RNA isolations, the *Arabidopsis* seedlings (10 DAG) on the 0.5 MS medium with 1% sucrose were transplanted to the same plate with different concentrations of AZD (0.5, 1, 2, and 5 μM) or DMSO. The shoots were harvested 1 day after transplanting to different AZD concentrations, or at different time points in the 2 μM AZD treatment (0, 24, 48, 72, 96 h) and then frozen in liquid nitrogen for RNA extraction. Total RNA was extracted with the RNAprep Pure Plant Kit (TianGen Biotech, Beijing, China) and measured using a DS-11 spectrophotometer (Denovix). The content of total RNA was measured by nanodrop through adding 1 μL RNA extractive uniformly.

### Sample preparation and cDNA library construction

Twenty-four hours after transplanting, three seedling plants were chosen randomly as one replicate and three replicates were used for each treatment, i.e., 2 μM AZD or DMSO. The RNA was treated with DNase I, and mRNA was enriched by Dynabeads mRNA Purification Kit (Life Technologies, #61006). The mRNA was fragmented to about 200 bp and the first strand of cDNA was synthesized using random hexamers. The buffer, dNTPs, RNase H, and DNA polymerase I were added to synthesize the second strand. The double strand cDNA was purified with magnetic beads followed by end repair and 3′-end single nucleotide adenine (A) addition. Finally, sequencing adaptors were ligated to the fragments, which were enriched by PCR amplification. The quantity and quality of the sample library were assessed using an Agilent 2100 Bioanaylzer and ABI StepOnePlus Real-Time PCR System. The libraries were then sequenced using the Illumina HiSeqTM 2000.

### Illumina sequencing and data analysis

High quality reads were obtained by removing the adaptor sequences, reads with more than 10% unknown bases (N's), and low quality reads (50% of bases with a quality value ≤ 10). The clean reads were mapped to the reference and/or reference gene set using SOAP aligner/SOAP2 (Li et al., 2009) (a max. of two mismatches was allowed in alignments). The expression was calculated using the Reads Per kb per Million reads (RPKM) method (Mortazavi et al., 2008). “The absolute value of log_2_Ratio ≥ 1 and probability ≥ 0.8” was regarded as the threshold to determine the significance of differential gene expression. The clustered genes were assigned to biological processes based on Gene Ontology (GO) using the web tool DAVID bioinformatics resources 6.7 (http://david.abcc.ncifcrf.gov/home.jsp) (Huang et al., 2008, 2009). Significantly enriched GO terms in DEGs compared with the genomic background (corrected *p* < 0.05) were identified based on a hypergeometric test. KEGG pathway-based analysis was performed using Blastall software against the KEGG database (http://www.genome.jp/kegg). The significantly enriched metabolic pathways or signal transduction pathways in the DEGs were also identified by pathway enrichment analysis (corrected *p* < 0.05) (Kanehisa et al., 2008). Comparisons of the DEGs in the data presented by three previous studies (Ren et al., 2012; Caldana et al., 2013; Xiong et al., 2013) and our data were conducted based on the online Venny analysis (http://bioinfogp.cnb.csic.es/tools/venny_old/index.html) (Oliveros, 2007). The total DEGs between the RNAi line and WT included the 3- and 6-day treatments, except the different tendencies of the DEGs (Caldana et al., 2013). For consistency, we treated the Glucose-TOR activated genes as down-regulated DEGs and the Glucose-TOR repressed genes as up-regulated DEGs (Xiong et al., 2013). This was done because the DEGs of the three remaining studies were based on the TOR-inhibition lines compared with WT, while the DEGs of Xiong et al. (2013) were based on a comparison between the WT and the TOR-repression lines.

### Real-time quantitative RT-PCR (qRT-PCR)

Plant samples for qRT-PCR were grown under the same conditions as the Illumina samples. One day after transferring 10-d-old plants to 2 μM AZD, shoots were harvested and frozen in liquid nitrogen. Total RNA was isolated using the RNAprep Pure Plant Kit (TianGen Biotech). Reverse transcription was performed with 1 μg of total RNA using the PrimeScript RT Kit (TAKARA Biotech). The qRT-PCR assays using the TransStart Top Green qPCR Super Mix (TransGen Biotech) were conducted on a Bio-Rad CFX96 System. Primer sequences for qRT-PCR were designed using Primer premier five software and were listed in Supplementary Table 1.

## Results

### AZD8055 is the strongest inhibitor of TOR in *Arabidopsis*

**Figure 1:**
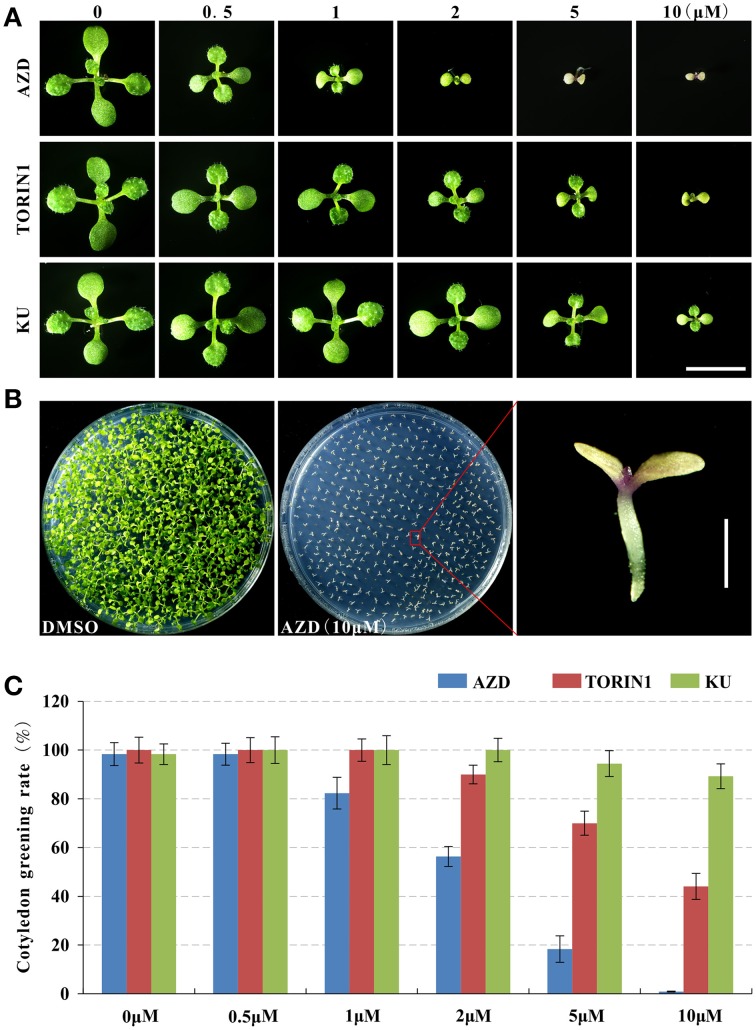
**asTORis efficiently inhibited cotyledon greening in ***Arabidopsis*****. Plants were grown on plates with different types of asTORis at different concentrations. **(A)** Cotyledons after 10 days growth with different inhibitors (AZD, TORIN1, and KU) at different concentrations. Bars = 1 cm. **(B)** Cotyledons after 10 days growth with 10 μM AZD and control DMSO. Bars = 2 mm. **(C)** Dose-dependent effect of AZD, TORIN1, and KU on the cotyledon greening rate after 10 days growth.

The inhibitory effects of AZD, TORIN1, and KU on plant growth were evaluated by germinating WT *Arabidopsis* seeds on plates with TOR inhibitors for 10 days. The growth of *Arabidopsis* was gradually retarded with the increasing concentrations of asTORis (Figure 1A). A dosage of 0.5 μM AZD was sufficient to inhibit seedling growth, followed by TORIN1 (1 μM) and KU (2 μM). The cotyledon greening rate and rosette leaf expansion was significantly inhibited by asTORis in a dose-dependent manner (Figures 1A–C). Severe defects in cotyledon greening occurred at 2 μM AZD, 5 μM TORIN1, and 10 μM KU. AZD was able to completely block the process of de-etiolation at 5 μM. Consistent with previous observations (Montané and Menand, 2013), the fresh weight and root length decreased with increasing doses of AZD, TORIN1 and KU, and AZD expressed the strongest inhibitory effects compared with TORIN1 and KU at the early stages of *Arabidopsis* (Supplementary Figures 1A–H), reflecting that TOR is instrumental to the early stages of plant growth, as has been observed in yeast and animals (Wullschleger et al., 2006). These results indicate that plants fail to establish photoautotrophic growth during the seed-to-seedling transition stage when TOR is inhibited. To further assess the functions of TOR in the post-seedling stage, 10 DAG seedlings were transferred from 0.5 × MS medium to 0.5 × MS medium supplemented with an asTORi and grown for 5 days. Consistent with the seed-to-seedling stage, the shoot fresh weight and primary root length of 10 DAG plants decreased gradually with increasing concentrations of AZD, TORIN1, and KU. It should be noted that AZD also displayed the strongest inhibitory effects at the post-seedling stage in *Arabidopsis* (Supplementary Figures 2A,B). AZD was therefore selected for the downstream expression analysis, but the optimal concentration and time of AZD treatment need to be determined. Since a previous study showed that TOR is a key player in regulating the content of total RNA in *Arabidopsis* (Ren et al., 2012), we measured the changes of total RNA content in response to different concentration and time of AZD treatment. Higher AZD concentrations resulted in a lower total RNA content in 10 DAG plants after growing on the medium with AZD for 1 day (Supplementary Figure 2C). Importantly, a 50% reduction of total RNA was apparent at 2 μM AZD. Furthermore, in *Arabidopsis* treated with 2 μM AZD, the turning point between the green and yellow cotyledon was observed, indicating that this concentration could maintain AZD specificity to TOR while maximizing DEGs for the RNA-seq experiments (Figure 1). Likewise, Longer treatment time lead to a lower total RNA when the 10 DAG plants were treated with 2 μM AZD, and the total RNA content also decreased 50% in 10 DAG seedlings treated with 2 μM AZD for 24 h (Supplementary Figure 2D), indicating that these parameters are the optimal time and dosage of AZD in *Arabidopsis* seedling treatment to minimize the secondary/indirect effects of the drug. We therefore used the samples of 10 DAG seedlings treated with 2 μM AZD for 24 h to perform RNA-seq experiments.

### Analysis of the transcriptional effects of TOR inhibition

**Figure 2:**
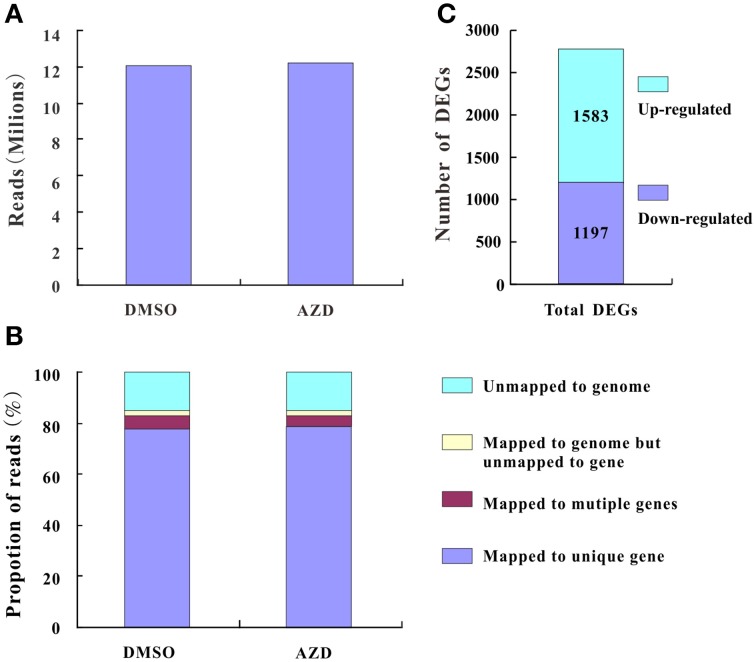
**The summary of basic information for the RNA-seq data. (A)** High-quality clean reads from high-throughput sequencing. **(B)** Proportions of high-quality clean reads of unmapped and/or mapped to unique genes, multiple genes, and the genome. **(C)** The number of differentially expressed genes.

After trimming for quality and adapter sequences, 12.21 and 12.04 million RNA-seq reads were obtained under the treatment of AZD and DMSO as control, respectively (Figure 2A), of which 85.10, 83.01, and 78.35% reads could be mapped to the annotated *Arabidopsis* genome, genes, and unigenes, respectively (Figure 2B). Between the AZD and DMSO control treatments, 2780 DEGs were found out of 24,347 genes detected (Supplementary Table 2A); 1583 were up-regulated and 1197 were down-regulated (Figure 2C). All of the DEGs were annotated in the NCBI NR database (http://blast.ncbi.nlm.nih.gov/Blast.cgi), the *Arabidopsis* information resource (TAIR) website (https://www.arabidopsis.org/), the DAVID bioinformatics resources 6.7 (http://david.abcc.ncifcrf.gov/home.jsp) or in other research results (Ren et al., 2012; Caldana et al., 2013; Xiong et al., 2013) (Supplementary Table 2B). The most up-regulated gene was the tumor necrosis factor receptor-associated factor (TRAF)-like family protein (396.18-fold), followed by a gene encoding “Major facilitator superfamily protein” (50.56-fold). Among the 15 most down-regulated genes, there were three genes encoding the SAUR-like auxin-responsive protein family (451.94-, 45.89-, and 37.53-fold) and two genes encoding an expansin family protein (35.75- and 34.76-fold), which play a critical role in cell wall modifications (Supplementary Figure 3).

One thousand and three hundred thirty three DEGs (47.95%) were assigned to one or more of three categories: biological process (BP), cellular component (CC), and molecular function (MF) base on the GO assignments (Huang et al., 2008, 2009). Further categorization resulted in the identification of 404 different GO terms and their enrichment (corrected *P* < 0.05) (Supplementary Table 3). The top three enriched GO terms were “cytosolic ribosome,” “structural constituent of ribosome,” and “ribosome,” supporting the conserved function of TOR in ribosome biogenesis (Martin et al., 2004; Ren et al., 2011). A total of 96 KEGG pathways were detected and 19 were enriched (corrected *P* < 0.05) (Supplementary Table 4 and Supplementary Figure 4). The top six enriched KEGG pathways, i.e., “ribosome,” “biosynthesis of secondary metabolites,” “biosynthesis of unsaturated fatty acid,” “alanine, aspartate, and glutamate metabolism,” “nitrogen metabolism,” and “regulation of autophagy,” were significantly affected by TOR inhibition.

### DEGs related to cell growth

**Figure 3:**
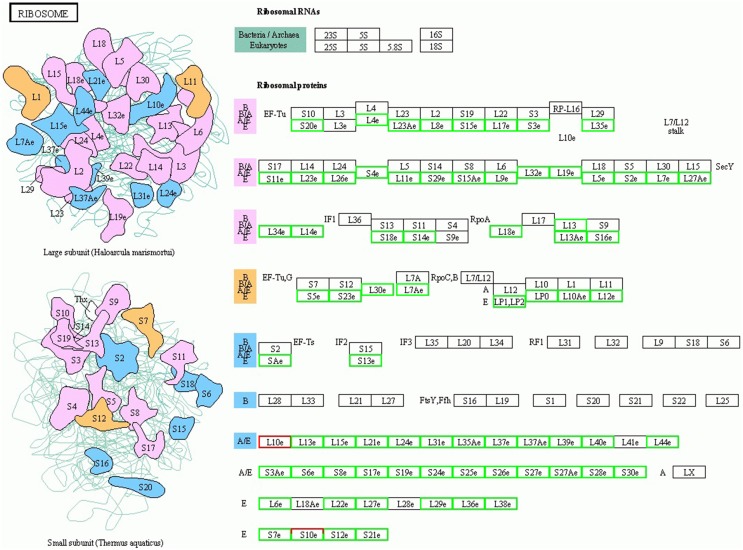
**TOR remodeled the gene expression profile of ribosome biosynthesis**. The schematic diagram of ribosome structure and its components are shown. Red boxes, up-regulated genes; green boxes, down-regulated genes.

Plant cell growth is tightly linked to ribosome biogenesis, cell wall expansin, and photosynthesis. The ribosome, composed of rRNAs and ribosomal proteins (RPs), is responsible for the synthesis of proteins in all cellular organisms (Ben-Shem et al., 2011). A differentially expressed rRNA gene was not detected in this study, likely due to the lack of a polyA tail. However, many DEGs associated with RP genes, including 114 down-regulated genes and 1 up-regulated gene, were assigned to the “ribosome pathway” (Figure 3 and Supplementary Table 5A), which was the most enriched pathway among the 96 KEGG pathways detected. Additionally, the regulation of ribosome biogenesis is a key component of cell growth control, and was also enriched in the GO biological processes (Supplementary Table 5B). Within this GO term, the genes encoding nucleolar proteins 1, 56, and 58 (NOP1, 56 and 58), U4/U6 small nuclear ribonucleoprotein (SNU13), and H/ACA ribonucleoprotein complex subunit four (DKC1) were down-regulated 3.03-, 2.14-, 2.00-, 3.03-, and 2.64-fold, respectively. These ribosomal core proteins combine with small nucleolar RNAs (snoRNAs, also downregulated) to form small nucleolar ribonucleoprotein particles (snoRAPs) that play a crucial role in ribosome biogenesis by guiding the processing and modification of pre-ribosomal RNA.

Cell wall elongation and expansin is another limiting factor for cell growth (Martin et al., 2001). In the classic model of cell wall elongation, expansin proteins loosen the cell wall by permitting the microfibril matrix network to slide. Then, the xyloglucan (XG) backbone is cut by the xyloglucan endotransglycosylase/hydrolase (XTE/XTH) and the fragments are transferred either to another XG or to water. Consequentially, the monosaccharides are connected to the end of the polysaccharides to synthesize the cellulose chains by cellulose synthase (CESA). In this study, a total of 46 DEGs were categorized in the GO-BP “cell wall organization” term (Supplementary Table 6A). For example, nine alpha-expansin genes and one beta-expansin gene were uniformly down-regulated by a 10.41- to 39.12-fold change, respectively, and 11 XTH and 3 CSL (CESA like) genes were differentially regulated. It is noteworthy that 9 DEGs were observed in cell wall thickening and 12 DEGs were detected in cell wall loosening (Supplementary Tables 6B,C).

**Figure 4:**
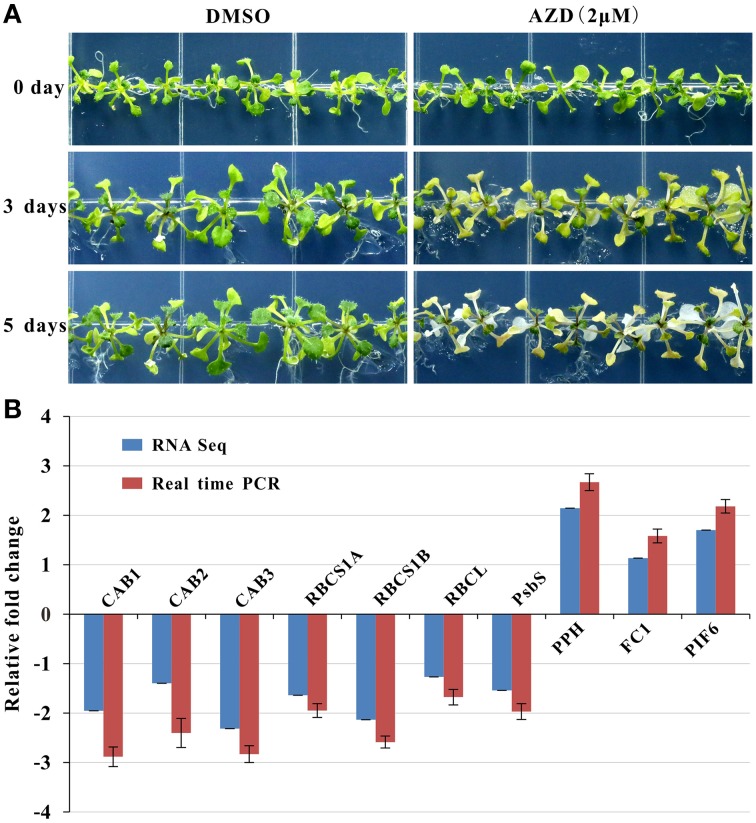
**AZD induced photosynthesis-related phenotypic and molecular changes in ***Arabidopsis*****. Plants were grown for 10 days without TOR inhibitors or DMSO, and then transferred to plates with TOR inhibitors and DMSO as the control. **(A)** Seedlings treated with AZD (2 μM) on 0, 3, and 5 days after transplanting. **(B)** Gene expression levels of photosynthesis-related genes in the 2-μM AZD treatment for 1 day growth after transplanting compared with the DMSO treatment both in the real time PCR and RNA-seq data. The genes associated with the Chlorophyll A/B binding proteins (CAB), Rubisco small subunit (RBCS), Rubisco large subunit (RBCL), Photosystem II subunit S (PSBS), Pheophytinase (PPH), Ferrochelatase I (FC1), and Phytochrome interacting factor 6 (PIF6) were examined.

Photosynthesis functions as one of the most important anabolic processes in plants, and total of 79 photosynthesis-associated DEGs were detected in this study (Table 1). Thirty DEGs were enriched in the KEGG “carbon fixation in photosynthetic organisms” pathway (Supplementary Figure 5). Of these 30 genes, two thirds of the DEGs were down-regulated. For example, four genes encoding the rate-limiting enzyme Rubisco small subunit (RBCS) family protein were uniformly down-regulated from a 3.14- to a 4.47-fold change. With regard to the light signaling pathway, there were 32 down-regulated marker genes with fold changes ranging from 2.10 to 5.43. One of the three up-regulated genes (AT5G13800) is involved in chlorophyll breakdown. In addition, 13 down-regulated genes and 1 up-regulated gene were assigned to chlorophyll biosynthesis. Consistently, the leaf etiolating occurred when 10 DAG seedlings were subjected to 2 μM AZD treatment for 3 days and the entire seedlings were bleached after 5 days AZD treatment (Figure 4A), indicating that the DEGs related to the photosynthesis and chloroplast were coupled with leaf color. To further confirm these observations, 10 key marker genes for chloroplast biogenesis and photosynthesis were selected for real-time PCR. Seven positive regulators of the chloroplast were significantly down-regulated while three negative regulators of photosynthesis were up-regulated (Figure 4B) after 24 h treatment with 2 μM AZD. These observations demonstrated that TOR plays a crucial role in chloroplast formation and photosynthesis in the post-seedling stage of *Arabidopsis* and provided insight into TOR signaling during plant development.

**Table 1:** **The DEGs related to photosynthesis in ***Arabidopsis*** under the condition of TOR inhibition with AZD**.

**GeneID**	**Putative function**	**log2FC**	**Probability**
**LIGHT REACTION**			
AT3G21055	Photosystem II subunit T (PSBTN)	−1.63	0.83
AT4G21280	Photosystem II subunit QA (PSBQA)	−1.61	0.82
AT1G44575	Photosystem II subunit S (PSBS)	−1.59	0.81
AT1G06680	Photosystem II subunit P-1 (PSBP-1)	−1.07	0.85
AT4G28660	Photosystem II reaction center PSB28	−1.59	0.89
AT1G67740	Photosystem II core complex PSBY	−1.08	0.85
AT1G05385	Low PSII accumulation 19, LPA19, PSB27-H1	−1.16	0.81
AT1G71500	Photosystem B protein 33, PSB33	−1.03	0.84
AT1G15820	Light harvesting complex photosystem II subunit 6 (LHCB6)	−1.53	0.81
AT5G54270	Light-harvesting chlorophyll B-binding protein 3 (LHCB3)	−1.07	0.85
AT5G11450	Mog1/PsbP/DUF1795-like photosystem II reaction center PsbP family	−1.56	0.81
AT5G27390	Mog1/PsbP/DUF1795-like photosystem II reaction center PsbP family protein	−1.64	0.81
ATCG00210	Electron transporter, transferring electrons within cytochrome b6/f complex of photosystem II activity (YCF6)	−2.44	0.83
AT5G44650	YCF3-interacting protein 1, Y3IP1	−1.25	0.85
AT1G52230	Photosystem I subunit H-2 (PSAH-2)	−1.32	0.87
AT3G16250	Photosynthetic NDH subcomplex B3 (PNSB3)	−1.10	0.84
AT1G19150	Photosystem I light harvesting complex gene 6 (LHCA6)	−1.68	0.83
AT1G49975	Involved in photosynthesis	−1.25	0.85
AT1G55370	NDH-dependent cyclic electron flow 5 (NDF5)	−1.07	0.81
AT1G29930	Chlorophyll A/B binding protein 1 (CAB1)	−1.98	0.84
AT1G29920	Chlorophyll A/B binding protein 2 (CAB2)	−1.40	0.86
AT1G29910	Chlorophyll A/B binding protein 3 (CAB3)	−2.34	0.86
AT4G17600	Light-harvesting chlorophyll a/b-binding (LHC) proteins (LIL3:1)	−1.50	0.81
AT5G13800	Pheophytinase (PPH) involved in chlorophyll breakdown	2.14	0.91
AT5G35220	Ethylene-dependent gravitropism-deficient and yellow-green 1 (EGY1)	−1.12	0.83
AT1G02280	Translocon at the outer envelope membrane of chloroplasts 33 (TOC33)	−1.37	0.86
AT3G46740	Translocon at the outer envelope membrane of chloroplasts 75-III (TOC75-III)	−1.55	0.81
AT4G03320	Translocon at the inner envelope membrane of chloroplasts 20-IV (TIC20-IV)	2.88	0.87
AT5G16620	Translocon at the inner envelope membrane of chloroplasts 40 (TIC40)	−1.15	0.83
AT3G52380	Chloroplast RNA-binding protein 33 (CP33)	−1.83	0.84
AT1G09340	Chloroplast RNA binding (CRB)	−1.65	0.83
AT5G49910	Chloroplast heat shock protein 70-2 (CPHSC 70-2)	−1.17	0.86
AT1G32080	LrgAB/CidAB protein involved in chloroplast development	−1.20	0.85
AT3G51890	Clathrin light chain 3 (CLC3)	1.73	0.83
AT1G79850	Pigment defective 347 (PDE347)	−1.49	0.81
**CARBON FIXATION**			
AT2G01290	Ribose-5-phosphate isomerase 2 (RPI2)	−1.01	0.82
AT1G71100	Ribose 5-phosphate isomerase	1.13	0.84
AT3G04790	Ribose 5-phosphate isomerase, type A protein	−1.50	0.88
AT2G01290	Ribose 5-phosphate isomerase	−1.01	0.82
AT3G55800	Sedoheptulose-1,7-bisphosphatase (SBPase)	−1.31	0.87
AT4G11280	1-Aminocyclopropane-1-carboxylate (ACC) synthase 6 (ACS6)	2.04	0.90
AT2G21330	Fructose-bisphosphate aldolase 1 (FBA1);	−1.74	0.90
AT4G38970	Fructose-bisphosphate aldolase 2 (FBA2)	−1.15	0.86
AT4G26530	Fructose-bisphosphate aldolase 5 (FBA5)	−2.11	0.91
AT4G26520	Fructose-bisphosphate aldolase 7 (FBA7)	−1.35	0.84
AT3G54050	Fructose 1,6-bisphosphate phosphatase	−1.14	0.86
AT1G53240	Mitochondrial malate dehydrogenase 1 (MMDH1)	−1.80	0.89
AT3G12780	Phosphoglycerate kinase 1 (PGK1)	−1.14	0.86
AT5G11520	Aspartate aminotransferase 3 (ASP3)	1.02	0.83
AT5G11670	NADP-malic enzyme 2 (NADP-ME2)	1.53	0.88
AT5G56350	Pyruvate kinase family protein	1.31	0.85
AT1G06570	4-hydroxyphenylpyruvate dioxygenase	2.14	0.91
AT4G15530	Pyruvate orthophosphate diskinase (PPDK)	2.69	0.93
AT4G21210	Pyruvate orthophosphate diskinase (PPDK)	−1.25	0.86
AT1G21440	Phosphoenolpyruvate carboxylase family protein	−2.18	0.90
AT5G17380	Thiamine pyrophosphate dependent pyruvate decarboxylase family protein	1.47	0.88
AT5G54960	Pyruvate decarboxylase-2 (PDC2)	1.62	0.89
AT3G60750	Transketolase 1 (TKL1)	−1.13	0.86
AT1G67090	Rubisco small subunit 1A (RBCS1A)	−1.65	0.83
AT5G38430	Rubisco small subunit 1B (RBCS1B)	−2.16	0.85
AT5G38420	Rubisco small subunit 2B (RBCS2B)	−1.84	0.84
AT5G38410	Rubisco small subunit 3B (RBCS3B)	−1.67	0.83
AT2G39730	Rubisco activase (RCA)	−1.75	0.90
ATCG00490	Rubisco large subunit (RBCL)	−1.27	0.82
AT3G62090	Phytochrome interacting factor 6 (PIF6)	1.70	0.82
**CHLOROPHYLL BIOSYNTHESIS**			
AT4G27440	Protochlorophyllide oxidoreductase B (PORB)	−2.11	0.91
AT5G08280	Porphobilinogen deaminase (PBGD)	−1.54	0.88
AT3G59400	Genomes uncoupled (GUN4)	−1.40	0.86
AT3G48730	Glutamate-1-semialdehyde 2,1-aminomutase 2 (GSA2)	−1.76	0.88
AT2G40490	Uroporphyrinogen decarboxylase (HEME2)	−1.62	0.88
AT3G14930	Uroporphyrinogen decarboxylase (HEME1)	−1.38	0.85
AT5G45930	Second Chl I gene (CHLI2)	−1.37	0.85
AT5G63570	Homology to glutamate-1-semialdehyde 2,1-aminomutase (GSA1)	−1.32	0.86
AT3G56940	Copper response deffect 1(CRD1)	−1.11	0.85
AT3G51820	Chlorophyll synthase (CHLG)	−1.44	0.86
AT1G03475	Oproporphyrinogen III oxidase (HEMF1)	−1.31	0.86
AT5G13630	Genomes uncoupled (GUN5)	−1.28	0.86
AT4G25080	Magnesium-protoporphyrin IX methyltransferase (CHLM)	−1.15	0.85
AT5G26030	Ferrochelatase I (FC1)	1.12	0.83

### DEGs involved in the regulation of autophagy and ubiquitination

**Figure 5:**
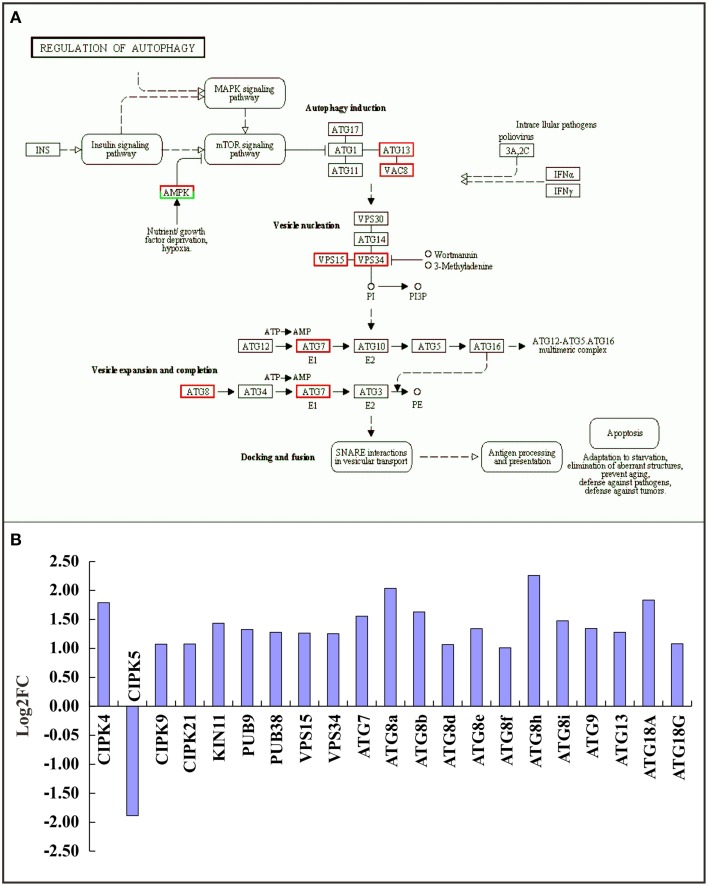
**Relationship between TOR and the autophagy pathway. (A)** Expression changes of genes in the autophagy pathway. Red boxes, up-regulated genes; green boxes, down-regulated genes. **(B)** The DEGs involved in the autophagy pathway. The genes encoding CBL-interacting protein kinase (CIPK), SNF1 kinase homolog (KIN), Plant u-box (PUB), Vacuolar protein sorting (VPS), and Autophagy (ATG) were detected.

Autophagy is a lysosome-dependent pathway for the turnover and recycling of intracellular large macromolecules and whole organelles. In this study, the “regulation of autophagy” KEGG pathway was one of the most enriched pathways in the RNA-seq data (Figure 5A and Supplementary Table 4). A total of 21 differentially expressed autophagy-associated genes were observed under TOR inhibition and most were significantly up-regulated (Figure 5B). For example, the genes encoding vacuolar protein sorting 15 and 34 (VPS15 and 34) were markedly up-regulated; these are protein kinases essential for autophagosome formation. Our results strongly support the previous observations in which TOR negatively regulated autophagy in yeast and animals (Liu and Bassham, 2010; Perez-Perez et al., 2010), suggesting that the interconnections between TOR signaling and autophagy are evolutionarily conserved across eukaryotic species.

The ubiquitin (Ub)/26S proteasome system (UPS) is involved in protein recycling in the cell. In the UPS, specific protein substrates are ubiquitinated and then degraded by the proteasome. Ub is covalently attached to target proteins through the sequential action of three enzymes: ubiquitin-activating enzyme (E1), ubiquitin-conjugating enzyme (E2), and ubiquitin-protein ligase (E3). The KEGG pathway “ubiquitin mediated proteolysis” was affected by the treatment of AZD (Supplementary Figure 6 and Supplementary Table 7). A total of 58 DEGs related to ubiquitination were detected, including 48 up-regulated and 10 down-regulated genes. Nine DEGs encoding E2 conjugating enzymes were up-regulated from fold changes of 2.15–3.48. Fourty-two DEGs (35 up-regulated and 7 down-regulated genes) have relationships with E3 ubiquitin-protein ligase. The DEGs related to the UPS showed that this process was activated under TOR inhibition, which opened the door to investigate the genetic links between TOR signaling and ubiquitination in future research.

### DEGs participated in phytohormone signaling pathways

Plant hormones are small molecular products that regulate many plant developmental processes at low concentrations (Rubio et al., 2009). We mainly focused on eight types of phytohormones, including auxin, gibberellin (GA), cytokinin (CK), brassinosteroid (BR), salicylic acid (SA), abscisic acid (ABA), ethylene (ET), and jasmonic acid (JA). Although the detailed mechanisms of how hormones modulate plant growth and development are far from being completely understood, the key components of all classic plant hormone pathways have been well characterized. We therefore sorted the DEGs for the phytohormone-related genes according to gene function, such as upstream signaling effectors, downstream responsive genes, or hormone transporters (Table 2). A total of 94 DEGs (59 up-regulated and 35 down-regulated genes) were identified to be involved in all eight phytohormone signal transduction pathways. The number of DEGs in the auxin signal transduction pathway was the highest (36 DEGs), followed by the ABA signal transduction pathway (19 DEGs), and the BR signal transduction pathway was the fewest (2 DEGs). Of the 59 up-regulated genes, the fold changes varied from 2.00 (BZIP25, AT3G54620) to 19.43 (JAZ7, AT2G34600). Most of the up-regulated genes were involved in the ABA, ET, JA, and SA signal pathways. For example, in the ET signaling pathway, the genes encoding the ethylene response factor, the ethylene responsive element-binding factor, and the ethylene-forming enzyme were all up-regulated from 3.56- to 13.74-fold. Similarly, in the ABA signaling pathway, there were 18 DEGs that were up-regulated from 2.03- to 14.22-fold. Of the 35 down-regulated expressed genes, the gene (AT3G03820) encoding small auxin upregulated RNA 29 (SAUR29) had the highest fold change (630.35, Log_2_ratio = –9.30), while the gene (AT2G46690) encoding SAUR32 exhibited the lowest absolute fold change (2.22, Log_2_ratio = –1.15). Most of the down-regulated genes were involved in the auxin, CK, BR, and GA signal pathways. For example, in the auxin signal pathway, there were 22 down-regulated DEGs encoding SAUR gene families, auxin influx and efflux carriers, and auxin responsive transcription factors. To further confirm these observations, 21 key marker genes involved in the phytohormone signaling pathway were selected for real-time PCR, and the expression level were nearly the same as that in the RNA-seq data (Supplementary Figure 7). It is well known that TOR acts as the major sensor of growth factors in yeast and animals (Wullschleger et al., 2006), and here we provide some important initial clues in plants.

**Table 2:** **Differentially expressed genes related to plant hormone signal transduction pathways**.

**Gene ID**	**Putative function**	**log2FC**	***P*-value**
**AUXIN SIGNAL TRANSDUCTION**			
AT4G34770	Small auxin upregulated RNA 1 (SAUR1)	−2.14	0.81
AT4G34790	Small auxin upregulated RNA 3 (SAUR3)	−2.34	0.82
AT2G21210	Small auxin upregulated RNA 6 (SAUR6)	−3.43	0.90
AT4G38840	Small auxin upregulated RNA 14 (SAUR14)	−3.67	0.90
AT4G38860	Small auxin upregulated RNA 16 (SAUR16)	−3.70	0.91
AT5G18020	Small auxin upregulated RNA 20 (SAUR20)	−5.23	0.91
AT5G18030	Small auxin upregulated RNA 21 (SAUR21)	−5.49	0.92
AT5G18080	Small auxin upregulated RNA 24 (SAUR24)	−3.43	0.85
AT3G03820	Small auxin upregulated RNA 29 (SAUR29)	−9.30	0.90
AT4G00880	Small auxin upregulated RNA 31 (SAUR31)	−1.42	0.85
AT2G46690	Small auxin upregulated RNA 32 (SAUR32)	−1.15	0.84
AT2G45210	Small auxin upregulated RNA 36 (SAUR36)	1.98	0.83
AT1G16510	Small auxin upregulated RNA 41 (SAUR41)	2.20	0.82
AT4G34760	Small auxin upregulated RNA 50 (SAUR50)	−2.51	0.85
AT1G75580	Small auxin upregulated RNA 51 (SAUR51)	−2.19	0.82
AT5G50760	Small auxin upregulated RNA 55 (SAUR55)	3.41	0.89
AT3G53250	Small auxin upregulated RNA 57 (SAUR57)	−1.61	0.81
AT1G20470	Small auxin upregulated RNA 60 (SAUR60)	−2.03	0.81
AT1G29450	Small auxin upregulated RNA 64 (SAUR64)	−2.89	0.82
AT1G56150	Small auxin upregulated RNA 71 (SAUR71)	1.89	0.82
AT3G12830	Small auxin upregulated RNA 72 (SAUR72)	1.58	0.85
AT5G20820	Small auxin upregulated RNA 76 (SAUR76)	−4.48	0.82
AT4G37390	Auxin-responsive GH3 family protein (GH3.2)	2.44	0.81
AT4G27260	Auxin-responsive GH3 family protein (GH3.5)	2.66	0.87
AT3G59900	Auxin-regulated gene involved in organ size (ARGOS)	1.42	0.84
AT5G35735	Auxin-responsive family protein	1.10	0.85
AT2G33860	Auxin response transcription factor 3 (ARF3)	−1.41	0.80
AT2G21050	Like auxin resistant 2 (LAX2), auxin influx carrier	−2.66	0.83
AT2G38120	Auxin resistant 1 (AUX1), auxin influx transporter	−1.25	0.83
AT3G02875	IAA-leucine resistant 1 (ILR1)	1.36	0.81
AT1G51760	IAA-alanine resistant 3 (IAR3)	1.76	0.89
AT1G70940	PIN-formed 3 (PIN3), auxin efflux carrier family protein	−1.12	0.83
AT1G76520	PIN-likes 3 (PILS3), auxin efflux carrier family protein	1.60	0.89
AT2G17500	PIN-likes 5 (PILS5), auxin efflux carrier family protein	1.91	0.89
AT3G10870	Methyl esterase 17 (MES 17), Methyl IAA esterase	−1.78	0.80
AT5G54490	PINOID-binding protein 1 (PBP1)	1.29	0.81
**CYTOKININ (CK) SIGNAL TRANSDUCTION**			
AT3G16857	Response regulator 1 (ARR1)	1.16	0.84
AT4G16110	Response regulator 2 (ARR2)	2.13	0.83
AT1G10470	Response regulator 4 (ARR4)	−1.72	0.82
AT5G62920	Response regulator 6 (ARR6)	−4.34	0.88
AT1G19050	Response regulator 7 (ARR7)	−2.07	0.82
AT3G57040	Response regulator 9 (ARR9)	−2.28	0.82
AT3G61630	Cytokinin response factor 6 (CRF6)	1.21	0.83
**GIBBERELLIN (GA) SIGNAL TRANSDUCTION**			
AT1G75750	GAST1 protein homolog 1 (GASA1)	1.58	0.81
AT5G15230	GAST1 protein homolog 4 (GASA4)	−2.86	0.87
AT1G74670	GA-stimulated Arabidopsis 6 (GASA6)	−3.64	0.91
AT3G10185	Gibberellin-regulated GASA/GAST/Snakin family protein	−4.04	0.89
AT3G63010	GA insensitive dwarf 1B (GID1B), Probable gibberellin receptor GID1L2	1.67	0.81
AT1G02400	Gibberellin 2-oxidase 6 (GA2OX6)	2.38	0.80
**BRASSINOSTERIOD (BR) SIGNAL TRANSDUCTION**			
AT1G70210	CYCLIN D1 (CYCD1)	−3.27	0.87
AT4G34160	CYCLIN D3 (CYCD3)	−1.73	0.81
**ABSCISIC ACID (ABA) SIGNAL TRANSDUCTION**			
AT2G30020	Protein phosphatase 2C 25 (PP2C25)	1.02	0.82
AT4G33920	Protein phosphatase 2C family protein (PP2C)	2.00	0.84
AT1G07630	Protein phosphatase 2C like gene	1.15	0.80
AT4G28400	Protein phosphatase 2C family protein (PP2C)	1.25	0.82
AT4G31860	Protein phosphatase 2C family protein (PP2C)	1.42	0.86
AT5G59220	Protein phosphatase 2C family protein (PP2C)	1.62	0.85
AT5G59220	Highly ABA-induced PP2C gene 1 (HAI1)	1.66	0.80
AT5G08350	ABA-responsive protein-related	2.69	0.86
AT3G02140	ABI five-binding protein 2 family protein (AFP2)	2.16	0.84
AT5G13630	ABA-binding protein (ABAR)	−1.28	0.86
AT5G05440	Regulatory component of ABA receptor 8 (RCAR8)	1.65	0.82
AT3G45640	Mitogen-activated protein (MAP) kinase 3 (MPK3)	2.66	0.87
AT4G11330	Mitogen-activated protein (MAP) kinase 5 (MPK5)	1.86	0.81
AT1G01560	Mitogen-activated protein (MAP) kinase 11 (MPK11)	2.26	0.85
AT2G01450	Mitogen-activated protein (MAP) kinase 17 (MPK17)	1.25	0.85
AT1G73500	Mitogen-activated protein (MAP) kinase kinase 9 (MKK9)	1.66	0.82
AT4G08500	Mitogen-activated protein (MAP) kinase kinase kinase 1 (MAPKKK1)	1.43	0.84
AT5G67080	Mitogen-activated protein (MAP) kinase kinase kinase 19 (MAPKKK19)	3.83	0.88
AT3G55270	Mitogen-activated protein (MAP) kinase phosphatase 1 (MKP1)	1.17	0.84
**ETHYLENE (ET) SIGNAL TRANSDUCTION**			
AT1G05010	Ethylene-forming enzyme (EFE)	2.12	0.85
AT1G28370	ERF domain protein 11 (ERF11)	2.60	0.91
AT3G50260	Cooperatively regulated by ethylene and jasmonate 1 CEJ1)	3.48	0.90
AT3G23240	Ethylene response factor 1 (ERF1)	3.78	0.91
AT3G23150	Ethylene response factor 2 (ETR2)	3.84	0.94
AT5G61600	Ethylene response factor 104 (ERF104)	2.55	0.87
AT4G17500	Ethylene responsive element binding factor 1 (ERF-1)	2.74	0.87
AT5G47220	Ethylene responsive element binding factor 2 (ERF-2)	1.97	0.84
AT5G47230	Ethylene responsive element binding factor 5 (ERF-5)	1.83	0.83
AT4G17490	Ethylene responsive element binding factor 6 (ERF-6)	3.03	0.88
**SALICYLIC ACID (SA) SIGNAL TRANSDUCTION**			
AT1G22070	TGA1A-related gene 3 (TGA3)	1.42	0.85
AT1G77920	TGACG sequence-specific binding protein 7 (TGA7)	2.04	0.84
AT3G54620	BZIP transcription factor family protein 25 (BZIP25)	1.00	0.82
AT2G42380	BZIP transcription factor family protein 34 (BZIP34)	−2.51	0.84
AT1G42990	BZIP transcription factor family protein 60 (BZIP60)	1.87	0.83
AT3G58120	BZIP transcription factor family protein 61 (BZIP61)	−1.65	0.81
AT5G28770	BZIP transcription factor family protein 63 (BZIP63)	1.48	0.85
**JASMONIC ACID (JA) SIGNAL TRANSDUCTION**			
AT2G34600	Jasmonate-zim-domain protein 7 (JAZ7), TIFY5B	4.28	0.91
AT4G32570	TIFY domain protein 8 (TIFY8)	−1.21	0.82
AT1G19180	Jasmonate-zim-domain protein 1 (JAZ1), TIFY10A	1.74	0.89
AT3G50260	Cooperatively regulated by ethylene and jasmonate 1(CEJ1)	3.48	0.90
AT1G51760	Jasmonic acid responsive 3 (JR3)	1.76	0.89
AT3G55970	Jasmonate-regulated gene 21 (JRG21)	2.55	0.85
AT5G27280	Zim17-type zinc finger protein	1.80	0.89

## Discussion

**Figure 6:**
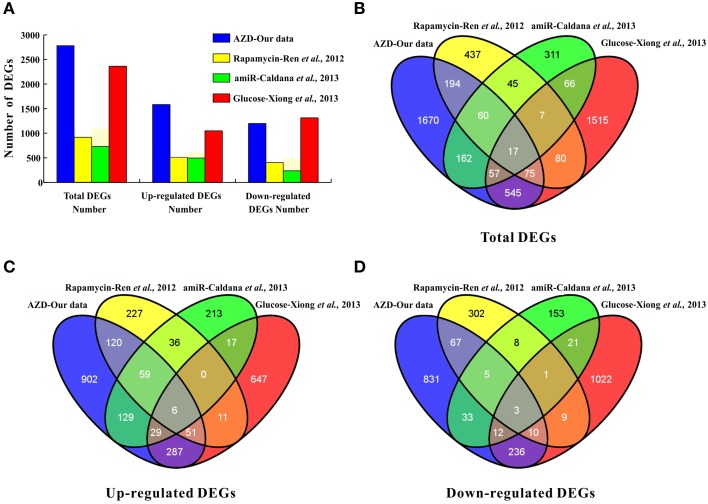
**Comparisons to other studies, i.e., (Ren et al., 2012; Caldana et al., 2013; Xiong et al., 2013). (A)** The number of DEGs. **(B)** The number of total DEGs shared among the four studies is represented by overlapping circles. **(C)** The number of up-regulated genes shared among the four studies is represented by overlapping circles. **(D)** The number of down-regulated genes shared among the four studies is represented by overlapping circles.

In *Arabidopsis*, the direct effects of rapamycin, KU, TORIN1, and AZD against TOR kinase activity were recently verified by independent groups (Ren et al., 2012; Montané and Menand, 2013; Schepetilnikov et al., 2013; Xiong et al., 2013). Montané and Menand (2013) clearly demonstrated that AZD acted in a TOR gene dose-dependent manner in *Arabidopsis* by combining pharmacological and genetic approaches. In addition, the GI50 of AZD (0.6 μM) was much lower than that of KU (3 μM) and WYE-354 (10 μM) (Montané and Menand, 2013). Furthermore, AZD was screened as the strongest asTORis in *Arabidopsis* compared with TORIN1 and KU (Figure 1 and Supplementary Figures 1, 2). Altogether, these results suggest that AZD is a more selective and potent TOR inhibitor than other asTORis. A total of 2780 DEGs were identified; 47.95% of the DEGs had at least one GO category, and 19 of the 96 KEGG pathways were enriched (Supplementary Tables 2–4 and Supplementary Figure 4). Compared with the previous three independent studies (Ren et al., 2012; Caldana et al., 2013; Xiong et al., 2013), the highest number of DEGs (2760) was identified in this study, followed by Xiong et al. (2362), Ren et al. (915), and Caldana et al. (725) (Figure 6A). This study shared the most overlapping DEGs (694) with those of Xiong et al. (2013), and 91% DEGs showed a similar tendency of expression changes (Figures 6B–D and Supplementary Table 8). A total of 346 overlapping DEGs were found between this study and those presented by Ren et al. (2012). 92.7% of these DEGs showed the identical tendency of expression changes, including 236 up-regulated and 85 down-regulated genes (Figures 6B–D and Supplementary Table 8). Two hundred and ninety-six DEGs were consistent with Caldana et al. (2013) and 276 out of 296 DEGs (93.2%) displayed the identical trend of expression changes (Figures 6B–D and Supplementary Table 8). The DEGs associated with the cell wall, ribosomes, and cell growth in this study was highly consistent with previous observations (Supplementary Table 9) (Ren et al., 2012; Caldana et al., 2013; Xiong et al., 2013). These results suggest that different approaches, conditions, materials, and treatments can result in similar expression (Moreau et al., 2012; Ren et al., 2012; Caldana et al., 2013; Xiong et al., 2013), indicating that TOR signaling is an evolutionarily conserved regulator of plant growth and development.

The activity of TOR is tightly regulated by growth factors in yeast and animals (Hsu et al., 2011; Xiong et al., 2013). Phytohormones are regarded as major plant growth factors. During the last decade, the metabolism, transport, perception, and signaling pathways of phytohormones and their regulation of plant growth, development, senescence and immune signaling network have been extensively studied (Rubio et al., 2009; Oliva et al., 2013; Vleesschauwer et al., 2014; Wang et al., 2015). However, the crosstalk between TOR signaling and phytohormones is largely unknown in plants. A recent study reported that TOR integrates auxin and nutrient signaling to regulate translation reinitiation and the selective translation of key transcriptional regulators (Schepetilnikov et al., 2013). TOR interacts with auxin and could thus interact with other phytohormones, such as CK, GA, BR, Eth, JA, ABA, and SA. Interestingly, upstream effectors and downstream response genes for all eight phytohormones were differentially expressed after 24 h of AZD treatment (Table 2). Most of the up-regulated genes were involved in the ABA, Eth, JA, and SA signal pathways, while most down-regulated genes were involved in the auxin, CK, BR, and GA signal pathways. This is in agreement with a common opinion that auxin, GA, CK, and BR are accelerators and the others are decelerators of shoot growth.

Taken together, the results from this study reveal that plants fail to establish photoautotrophic growth during the seed-to-seedling transition stage when TOR is inhibited by asTORis. TOR plays a crucial role in chloroplast formation and photosynthesis in the post-seedling stage in *Arabidopsis*. The expression analysis supports a conserved function of TOR in ribosome biogenesis, cell wall elongation, and autophagy, and provides new insights of the involvement of TOR in photosynthesis and phytohormone signaling. Thus, this study provides a platform to further study the downstream targets of TOR in *Arabidopsis*.

## Author contributions

MR, PD, and FX designed the experiments; PD, FX, YQ, KW, LY, ZL, and MR performed the experiments; PD, FX, and MR analyzed the data, and MR, PD, and FX wrote the manuscript.

### Conflict of interest statement

The authors declare that the research was conducted in the absence of any commercial or financial relationships that could be construed as a potential conflict of interest.

## Supplementary material

Supplementary Figure 1 **asTORis efficiently inhibited *Arabidopsis* shoot and root growth**. Plants were grown directly on plates with different types of asTORis at different concentrations. **(A–F)** Whole plants after 10 days growth on different concentrations of AZD. Bars = 1 cm. **(G)** Dose-response curves of AZD, TORIN1, and KU for shoot fresh weight after 10 days growth with inhibitors. **(H)** Dose-response curves of AZD, TORIN1, and KU for primary root length after growing for 10 days with inhibitors.

Supplementary Figure 2 **asTORis efficiently inhibited the seedling growth of *Arabidopsis***. Plants were grown for 10 days without TOR inhibitors or DMSO, and then transferred to plates with TOR inhibitors and DMSO as the control. **(A)** Dose-response curves of AZD, TORIN1, and KU for shoot fresh weight for 5 days growth after transplanting. **(B)** Dose-dependent effect of AZD, TORIN1, and KU on primary root length for 5 days growth after transplanting. **(C)** The RNA content of *Arabidopsis* seedlings treated with AZD at different concentrations for 1 day growth after transplanting. **(D)** The RNA content of *Arabidopsis* seedlings at different time points after transferring to the plates with AZD (2 μM).

Supplementary Figure 3 **The top 15 up- and down-regulated genes**. 1, TRAF-like family protein; 2, Major facilitator superfamily protein; 3, Peroxidase superfamily protein; 4, Integrase-type DNA-binding superfamily protein; 5, PEBP-like protein; 6, Proline-rich extensin-like family protein; 7, Peroxidase superfamily protein; 8, PLC-like phosphodiesterases superfamily protein; 9, Unknown protein; 10, Sugar transporter ERD6-like 16; 11, Cytochrome P450, family 81; 12, Senescence-associated gene 12; 13, Myb domain protein 90; 14, Myo-inositol oxygenase; 15, Proline dehydrogenase; 16, Expansin A15; 17, Expansin B3; 18, SAUR-like auxin-responsive protein family; 19, SAUR-like auxin-responsive protein family; 20, Alkeny hydroxalkyl producing 2; 21, Lipid transfer protein 4; 22, Anter-specific proline-rich protein APG; 23, Late embryogenesis abundant protein family protein; 24, Unknown protein; 25, EamA-like transporter family protein; 26, Myb domain protein 76; 27, Zinc-binding dehydrogenase family protein; 28, Myb domain protein 29; 29, Mto 1 responding down 1; 30, SAUR-like auxin-responsive protein family.

Supplementary Figure 4 **Scatter plot of KEGG pathway enrichment statistics from the *Arabidopsis* seedlings treated with AZD and DMSO**. Rich Factor, the ratio of the number of DEGs to the number of background genes in a KEGG pathway; corrected *Q* < 0.05 as the condition of enrichment.

Supplementary Figure 5 **The expression changes of the genes in the carbon fixation pathway in photosynthetic organisms**. Red boxes indicate up-regulated genes and green boxes represent down-regulated genes.

Supplementary Figure 6 **The expression changes of the genes in the ubiquitin mediated proteolysis pathway**. Red boxes indicate up-regulated genes and green boxes represent down-regulated genes.

Supplementary Figure 7 **Expression levels of phytohormone associated genes in the 2-μM AZD treatment for 24 h compared with the DMSO treatment**. Small auxin up-regulated RNA (SAUR), Response regulator (ARR), GA-stimulated Arabidopsis (GASA), CYCLIN D1 (CYCD1), ABA-responsive protein-related (ABR), ABI five-binding protein 4 family protein (AFP4), Protein phosphatase 2C family protein (PP2C), Mitogen-activated protein (MAP) kinase kinase kinase 19 (MAPKKK19), Ethylene response factor 1 (ERF1), Ethylene response factor 2 (ETR2), Cooperatively regulated by ethylene and jasmonate 1 (CEJ1).

Supplementary Table 1 **Primers used for real-time PCR**.

Supplementary Table 2 **(A)** The total detected genes in the RNA-seq data. **(B)** The total differentially expressed genes (DEGs) and their functions. The criteria of DEGs: the /log2 ratio/≥1 and the Probability value (*P*-value for hypothesis testing) of 0.8 or more.

Supplementary Table 3 **(A)** The Gene Ontology (GO)-cellular component assignments and their enrichment. **(B)** The Gene Ontology (GO)-molecular function assignments and their enrichment. **(C)** The Gene Ontology (GO)-biological process assignments and their enrichment.

Supplementary Table 4 **Summary of KEGG pathway in *Arabidopsis* seedling with AZD treatment**.

Supplementary Table 5 **(A)** The DEGs involved in the ribosome pathway (KEGG pathway). **(B)** The DEGs involved in the GO biological process term “ribosome biogenesis.”

Supplementary Table 6 **(A)** The DEGs involved in the GO biological process term “cell wall organization.” **(B)** The DEGs involved in the GO biological process term “cell wall thicking.” **(C)** The DEGs involved in the GO biological process term “cell wall loosing.”

Supplementary Table 7 **The DEGs involved in the KEGG Pathway “ubiquin mediated proteolysis**.”

Supplementary Table 8 **The number of the same genes with similar regulation tendency among our data and previous researches (Ren et al., 2012; Caldana et al., 2013 and Xiong et al., 2013)**.

Supplementary Table 9 **(A)** The same DEGs detected both in our data and Caldana et al. (2013). **(B)** The same DEGs detected both in our data and Ren et al. (2012). **(C)** The same DEGs detected both in our data and Xiong et al. (2013). **(D)** The same DEGs detected both in Caldana et al. (2013) and Xiong et al. (2013). **(E)** The same DEGs detected both in Ren et al. (2012) and Xiong et al. (2013). **(F)** The same DEGs detected both in Ren et al. (2012) and Caldana et al. (2013).
