# Supplementary figures and images for: Expression profiling and functional analysis reveals that TOR is a key player in regulating photosynthesis and phytohormone signaling pathways in Arabidopsis
Source: Front Plant Sci. 2015 Sep 7;6:677. doi: 10.3389/fpls.2015.00677 (PMC4561354; doi:10.3389/fpls.2015.00677)

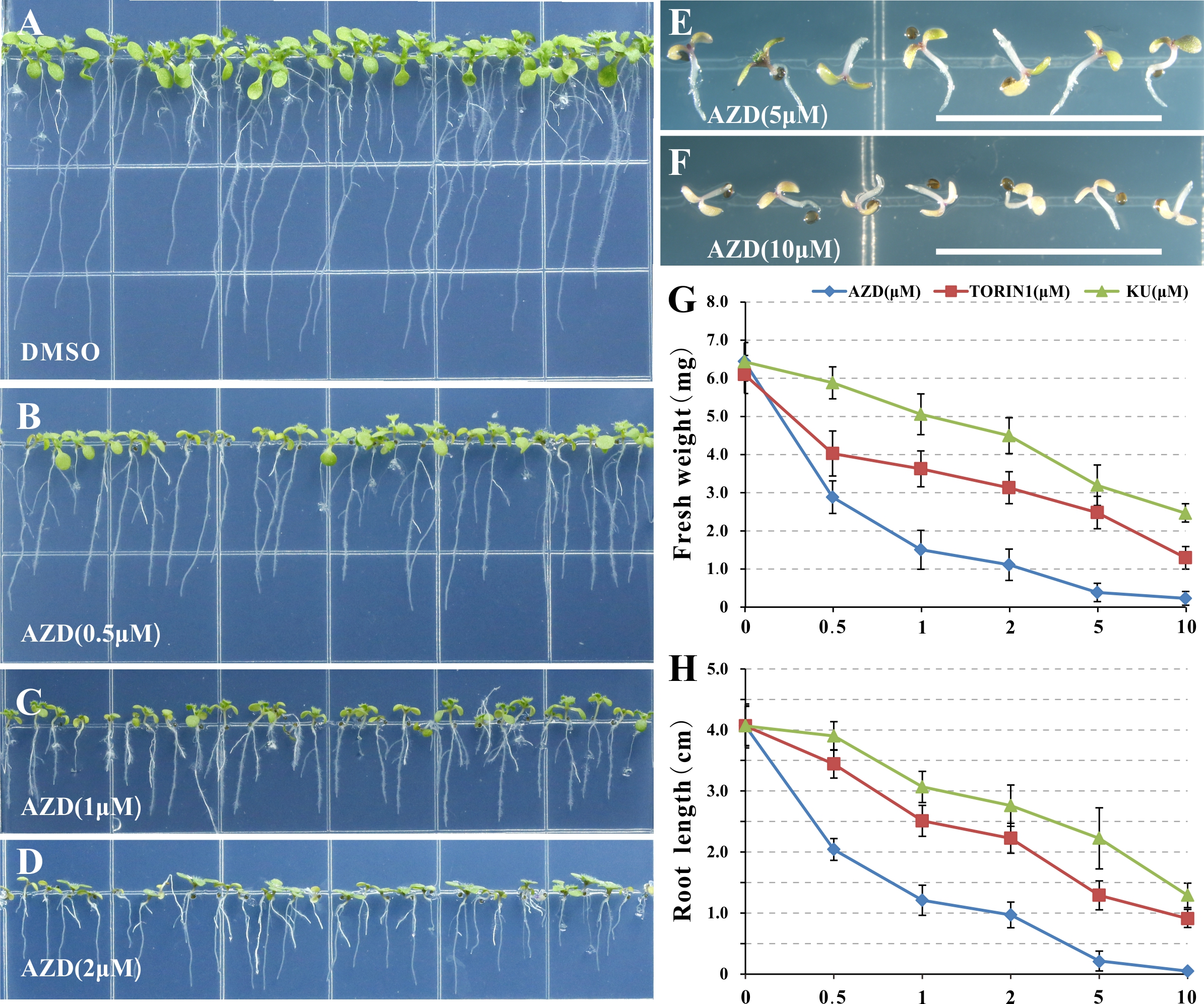

Supplement: Supplementary Figure 1 — asTORis efficiently inhibited Arabidopsis shoot and root growth. Plants were grown directly on plates with different types of asTORis at different concentrations. (A–F) Whole plants after 10 days growth on different concentrations of AZD. Bars = 1 cm. (G) Dose-response curves of AZD, TORIN1, and KU for shoot fresh weight after 10 days growth with inhibitors. (H) Dose-response curves of AZD, TORIN1, and KU for primary root length after growing for 10 days with inhibitors. [file Image1.JPEG]

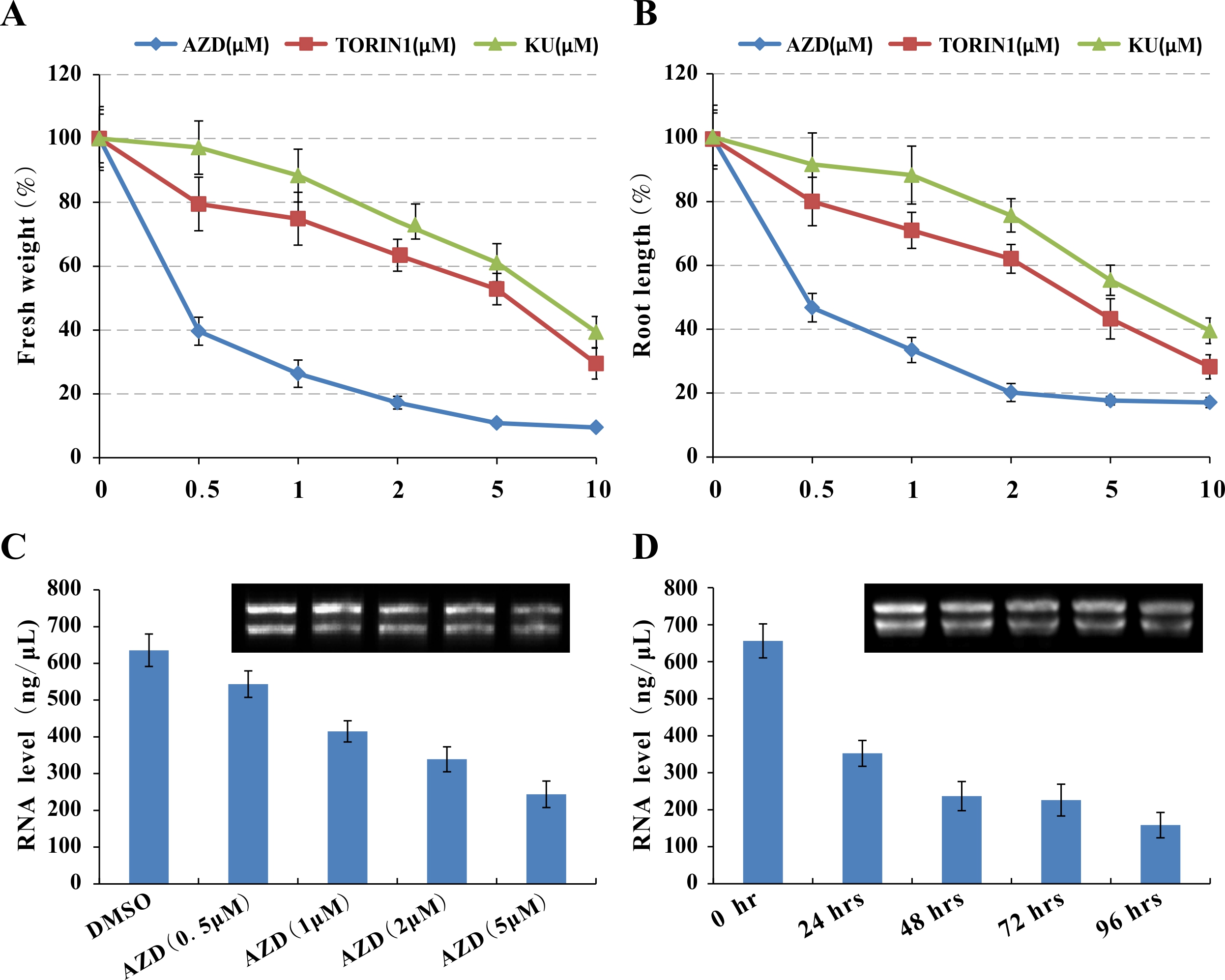

Supplement: Supplementary Figure 2 — asTORis efficiently inhibited the seedling growth of Arabidopsis. Plants were grown for 10 days without TOR inhibitors or DMSO, and then transferred to plates with TOR inhibitors and DMSO as the control. (A) Dose-response curves of AZD, TORIN1, and KU for shoot fresh weight for 5 days growth after transplanting. (B) Dose-dependent effect of AZD, TORIN1, and KU on primary root length for 5 days growth after transplanting. (C) The RNA content of Arabidopsis seedlings treated with AZD at different concentrations for 1 day growth after transplanting. (D) The RNA content of Arabidopsis seedlings at different time points after transferring to the plates with AZD (2 μM). [file Image2.JPEG]

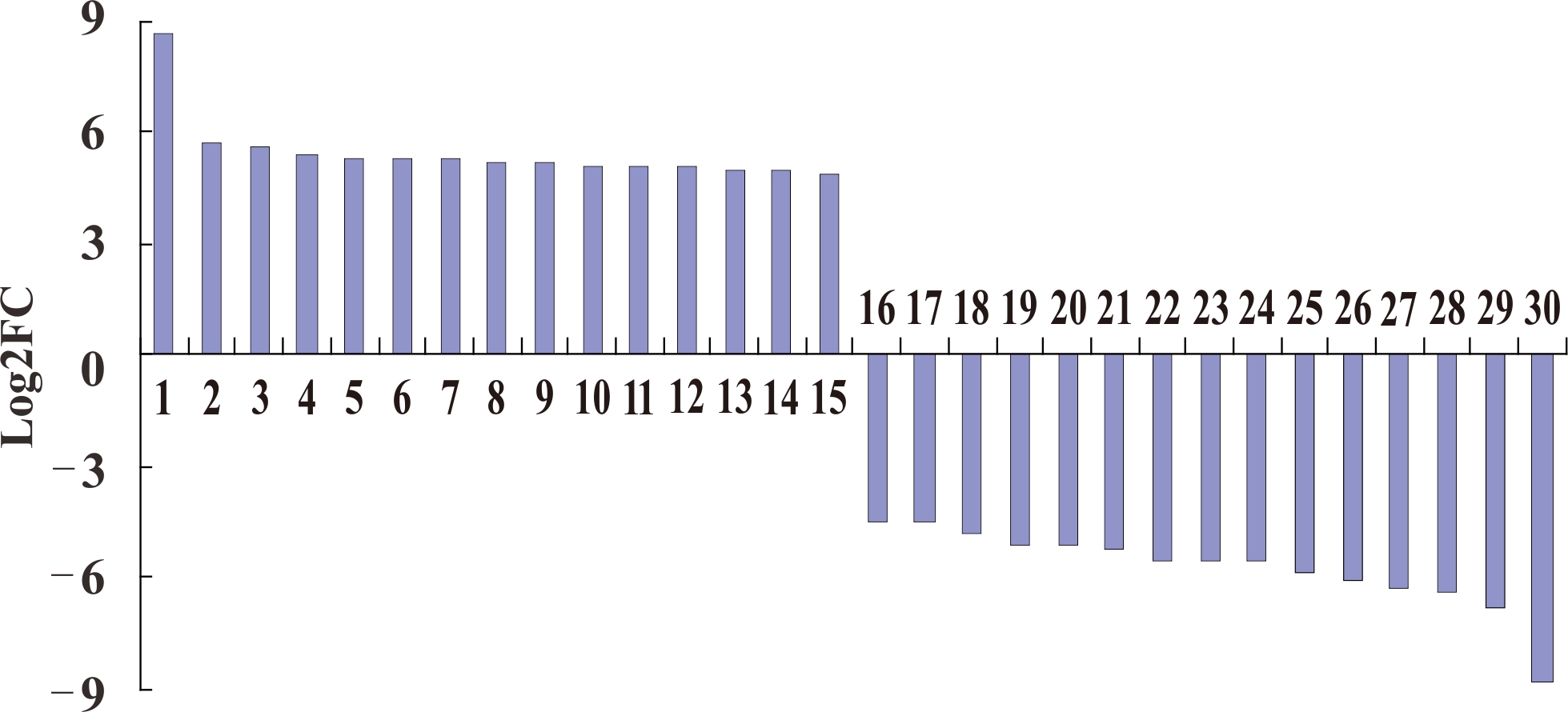

Supplement: Supplementary Figure 3 — The top 15 up- and down-regulated genes. 1, TRAF-like family protein; 2, Major facilitator superfamily protein; 3, Peroxidase superfamily protein; 4, Integrase-type DNA-binding superfamily protein; 5, PEBP-like protein; 6, Proline-rich extensin-like family protein; 7, Peroxidase superfamily protein; 8, PLC-like phosphodiesterases superfamily protein; 9, Unknown protein; 10, Sugar transporter ERD6-like 16; 11, Cytochrome P450, family 81; 12, Senescence-associated gene 12; 13, Myb domain protein 90; 14, Myo-inositol oxygenase; 15, Proline dehydrogenase; 16, Expansin A15; 17, Expansin B3; 18, SAUR-like auxin-responsive protein family; 19, SAUR-like auxin-responsive protein family; 20, Alkeny hydroxalkyl producing 2; 21, Lipid transfer protein 4; 22, Anter-specific proline-rich protein APG; 23, Late embryogenesis abundant protein family protein; 24, Unknown protein; 25, EamA-like transporter family protein; 26, Myb domain protein 76; 27, Zinc-binding dehydrogenase family protein; 28, Myb domain protein 29; 29, Mto 1 responding down 1; 30, SAUR-like auxin-responsive protein family. [file Image3.JPEG]

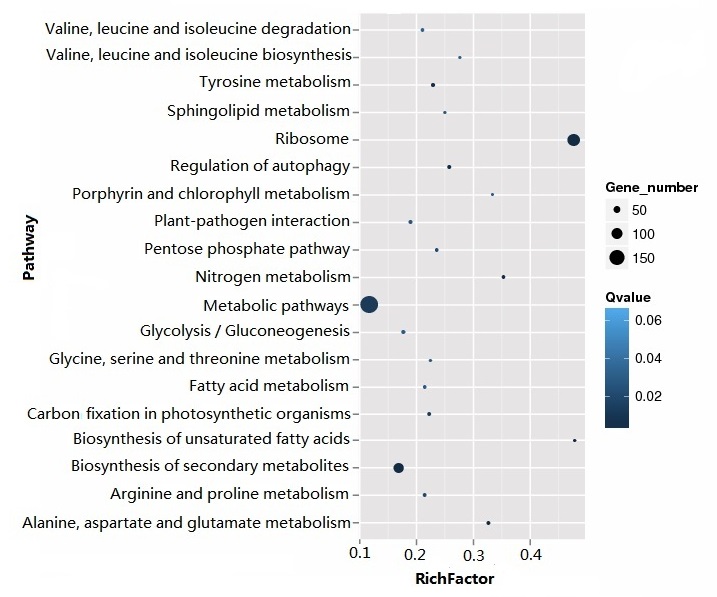

Supplement: Supplementary Figure 4 — Scatter plot of KEGG pathway enrichment statistics from the Arabidopsis seedlings treated with AZD and DMSO. Rich Factor, the ratio of the number of DEGs to the number of background genes in a KEGG pathway; corrected Q < 0.05 as the condition of enrichment. [file Image4.JPEG]

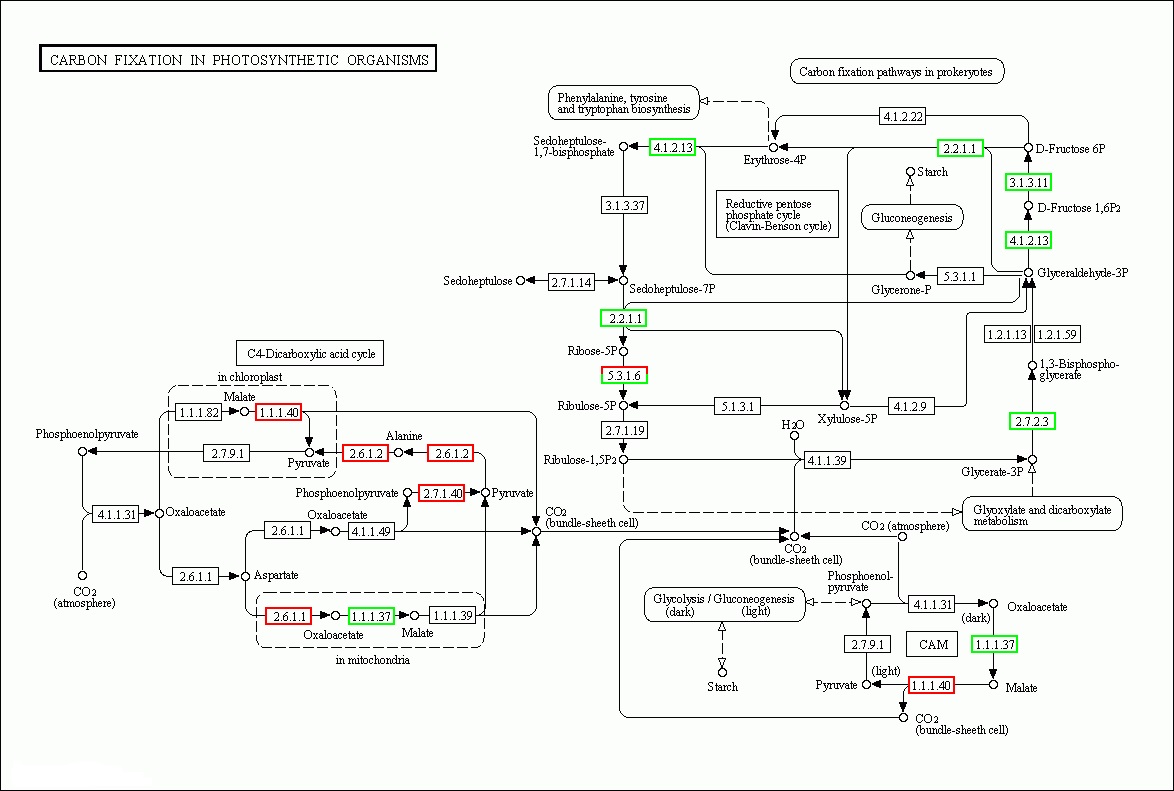

Supplement: Supplementary Figure 5 — The expression changes of the genes in the carbon fixation pathway in photosynthetic organisms. Red boxes indicate up-regulated genes and green boxes represent down-regulated genes. [file Image5.JPEG]

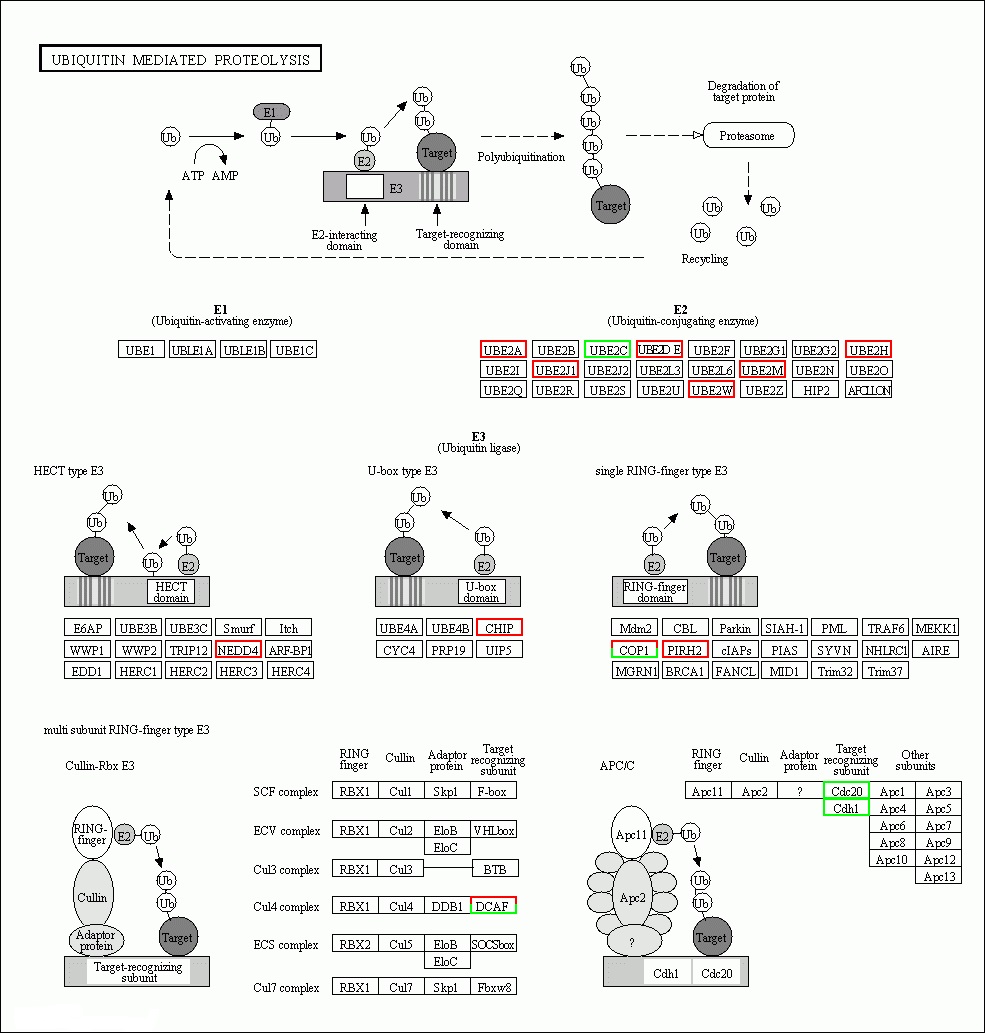

Supplement: Supplementary Figure 6 — The expression changes of the genes in the ubiquitin mediated proteolysis pathway. Red boxes indicate up-regulated genes and green boxes represent down-regulated genes. [file Image6.JPEG]

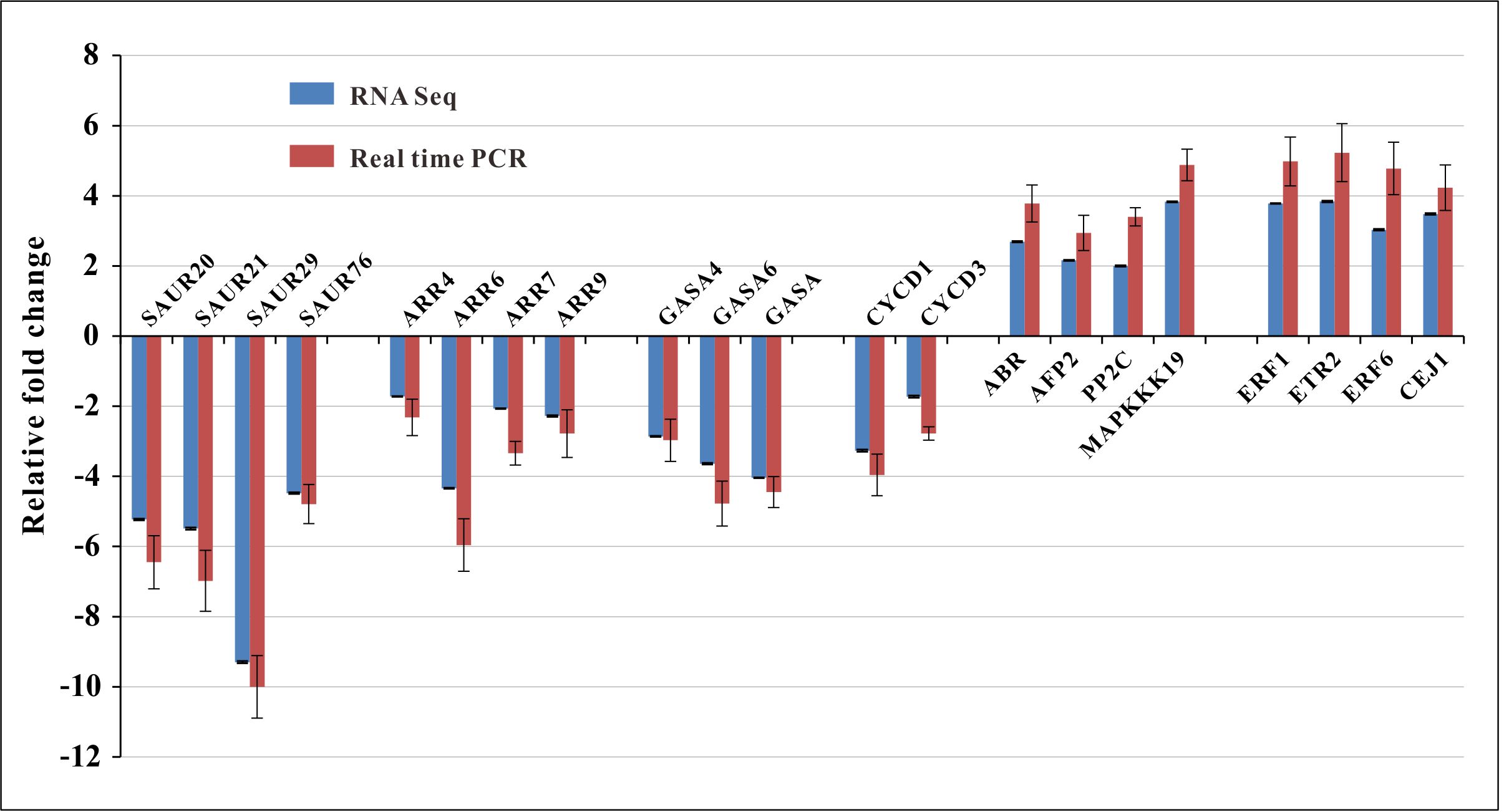

Supplement: Supplementary Figure 7 — Expression levels of phytohormone associated genes in the 2-μM AZD treatment for 24 h compared with the DMSO treatment. Small auxin up-regulated RNA (SAUR), Response regulator (ARR), GA-stimulated Arabidopsis (GASA), CYCLIN D1 (CYCD1), ABA-responsive protein-related (ABR), ABI five-binding protein 4 family protein (AFP4), Protein phosphatase 2C family protein (PP2C), Mitogen-activated protein (MAP) kinase kinase kinase 19 (MAPKKK19), Ethylene response factor 1 (ERF1), Ethylene response factor 2 (ETR2), Cooperatively regulated by ethylene and jasmonate 1 (CEJ1). [file Image7.JPEG]
