# Supplementary material for: Expression profiling and functional analysis reveals that TOR is a key player in regulating photosynthesis and phytohormone signaling pathways in Arabidopsis
Source: Front Plant Sci. 2015 Sep 7;6:677. doi: 10.3389/fpls.2015.00677 (PMC4561354; doi:10.3389/fpls.2015.00677)
Supplement: Supplementary Table 1 — Primers used for real-time PCR. [file Table1.DOCX]

Supplementary Table 1 Primers used for real-time PCR.

| Gene | Accession no. | Forward (5’-3’) | Reverse (5’-3’) |
| --- | --- | --- | --- |
| *CAB1* | AT1G29930 | TGAAGGCTACAGAGTCGCAGG | ACCAGTGACGATGGCTTGAACG |
| *CAB2* | AT1G29920 | TGAAGGTTACAGAGTCGCAG | AGTGACGATGGCTTGAACG |
| *CAB3* | AT1G29910 | GGACTTGCTTTACCCCGGTG | TCGGTAGCAAGACCCAATGG |
| *RBCS1A* | AT1G67090 | TATGGTCGCTCCTTTCAACG | TGATGCACTGGACTTGACGG |
| *RBCS1B* | AT5G38430 | CTCCCTGACCTTACTGACG | GCTTGTAGGCAATGAAACTG |
| *RBCSL* | ATCG00490 | AACTTGAAGGAGACAGGGAG | CTGAAGCCACAGGCAGAACA |
| *PsbS* | AT1G44575 | TGCTTACTTCAGGCGTCACC | AACCTTGCTCTTCGGCTTC |
| *PPH* | AT5G13800 | ATTATCGAGTATGGGCTATTG | CAGGTGAGGATGGGTTGC |
| *FC1* | AT5G26030 | TTAAGACGAATGTGTTTGAG | TCGGAGCACGAACAACAGA |
| *PIF6* | AT3G62090 | TGGTTGCTGATAGGTCGTTC | TTCGTTGTCGTCCTTGTGAG |
| *SAUR20* | AT5G18020 | ATTCTAAGCCGCTCCACCA | ATCGTTAAGCCACCCATTG |
| *SAUR21* | AT5G18030 | AGGGTTTCTTGCGGTGTAC | AGGACAAGGGATCGTTAAG |
| *SAUR29* | AT3G03820 | ATGGCTTTGGTAAGAGGT | AGGCTGGTTCAAGTATGA |
| *SAUR76* | AT5G20820 | ATGGCGAAAGGAGGAAAC | AGGATCGGCTGTGATTGG |
| *ARR4* | AT1G10470 | TTACGGCGGTAGATAGTGG | GTGGAGGAAGCGAAGAGT |
| *ARR6* | AT5G62920 | AGTTATGCTACCGAGGAAG | ACATCTATCAATACGAGGC |
| *ARR7* | AT1G19050 | ATTTGATAGTGACGGATTAC | TCTGCTCCTTCTTTGAGAC |
| *ARR9* | AT3G57040 | GTATGGCAGCAGAATCGC | AACCCAGAAACTCCAAAGC |
| *GASA4* | AT5G15230 | CGGAATGTGATAGGAGGTG | AGTTGTTGTAGCAGGAGCA |
| *GASA6* | AT1G74670 | TATTCACTTTCGTTTGTCTC | ACACTTGTTTGTTGCCGTAC |
| *GASA* | AT3G10185 | TTGTGCTGAATGCGAAAC | TCCACCGCTCTTGGTCTTC |
| *CYCD1* | AT1G70210 | AGAGCCCTGAGACTTGGT | CGATGATGCCCTTACACT |
| *CYCD3* | AT4G34160 | CAACAAATGCCACCGTCTC | TTCGTCGCTATTGAAAGG |
| *ABR* | AT5G08350 | CTTCAGGTTGGAGGTTTAG | TCGACCGTGACTACTTCTAG |
| *AFP4* | AT3G02140 | GAGACTCTGAGCATGGTGAG | GACCCAGTTCAAAGTGCCA |
| *PP2C* | AT4G33920 | ATAGTAATAAAGGTGCCG | TTTGTGAGTCATCAGGGT |
| *MAPKKK19* | AT5G67080 | GTGACGAGGTTCCGATGA | GACGGTTACAAATGGATGGT |
| *ERF1* | AT3G23240 | AGTCCACGCAACAAACCT | GAGCCAAACCCTAATACC |
| *ETR2* | AT3G23150 | GTGCCGTGGCTGCTATTAG | TCCTCGCTTGGCTCGCTCT |
| *ERF6* | AT4G17490 | GTCTCCGTTGCCTACTACTG | CCTCTGTAATGCCTCTTCTC |
| *CEJ1* | AT3G50260 | AAGACCATTCAAAGGGATC | GTAGAGTAAGAGCCGAGCC |
| *Actin* | AT3G18780 | GCACTTGCACCAAGCAGCAT | CCTTTCAGGTGGTGCAACGAC |
