# Supplementary material for: Expression profiling and functional analysis reveals that TOR is a key player in regulating photosynthesis and phytohormone signaling pathways in Arabidopsis
Source: Front Plant Sci. 2015 Sep 7;6:677. doi: 10.3389/fpls.2015.00677 (PMC4561354; doi:10.3389/fpls.2015.00677)
Supplement: Supplementary Table 8 — The number of the same genes with similar regulation tendency among our data and previous researches (Ren et al., 2012; Caldana et al., 2013 and Xiong et al., 2013). [file Table8.DOC]

Supplementary Table 8 The number of the same genes with similar regulation tendency among our data and previous researches (Ren et al., 2012, Caldana et al., 2013 and Xiong et al., 2013)

|  | Our data | Ren et al 2012 | Caldana et al 2013 | Xiong et al 2013 |
| --- | --- | --- | --- | --- |
| Our data |  | 85 | 53 | 261 |
| Ren et al 2012 | 236 |  | 17 | 23 |
| Caldana et al 2013 | 223 | 101 |  | 37 |
| Xiong et al 2013 | 373 | 68 | 52 |  |

Note: The number of the same genes with up-regulated tendency was in the bottom left corner (red). The number of the same genes with down-regulated tendency was in the top right corner (blue).
